# Role of klotho and fibroblast growth factor 23 in arterial calcification, thickness, and stiffness: a meta-analysis of observational studies

**DOI:** 10.1038/s41598-024-56377-8

**Published:** 2024-03-08

**Authors:** Citrawati Dyah Kencono Wungu, Hendri Susilo, Mochamad Yusuf Alsagaff, Bendix Samarta Witarto, Andro Pramana Witarto, Cennikon Pakpahan, Arief Gusnanto

**Affiliations:** 1 Department of Physiology and Medical Biochemistry, Division of Biochemistry, Faculty of Medicine, Universitas Airlangga, Surabaya, 60132 Indonesia; 2 Institute of Tropical Disease, Universitas Airlangga, Surabaya, 60115 Indonesia; 3 Department of Cardiology and Vascular Medicine, Faculty of Medicine, Universitas Airlangga, Surabaya, 60132 Indonesia; 4 Department of Cardiology and Vascular Medicine, Universitas Airlangga Hospital, Universitas Airlangga, Surabaya, 60115 Indonesia; 5 Medical Program, Faculty of Medicine, Universitas Airlangga, Surabaya, 60132 Indonesia; 6 Department of Biomedical Sciences, Faculty of Medicine, Universitas Airlangga, Surabaya, 60132 Indonesia; 7 School of Mathematics, University of Leeds, Leeds, LS2 9JT UK

**Keywords:** Arterial calcification, Arterial stiffness, Arterial thickness, Cardiovascular diseases, Fibroblast growth factor-23, Klotho, Biochemistry, Physiology, Cardiology, Medical research

## Abstract

This meta-analysis was conducted to clarify the role of klotho and fibroblast growth factor 23 (FGF-23) in human arterial remodeling across recent studies, in terms of arterial calcification, thickness, and stiffness. A systematic literature search was conducted on five databases for articles up to December 2023. Arterial calcification, thickness, and stiffness were determined using the calcification score and artery affected, carotid intima–media thickness (CIMT), and pulse wave velocity (PWV), respectively. Sixty-two studies with a total of 27,459 individuals were included in this meta-analysis. Most studies involved chronic kidney disease patients. Study designs were mostly cross-sectional with only one case–control and nine cohorts. FGF-23 was positively correlated with arterial calcification (r = 0.446 [0.254–0.611], p < 0.0001 and aOR = 1.36 [1.09–1.69], p = 0.006), CIMT (r = 0.188 [0.02–0.354], p = 0.03), and PWV (r = 0.235 [0.159–0.310], p < 0.00001). By contrast, Klotho was inversely correlated with arterial calcification (r = − 0.388 [− 0.578 to − 0.159], p = 0.001) and CIMT (r = − 0.38 [− 0.53 to − 0.207], p < 0.00001). In conclusion, FGF-23 and Klotho were associated with arterial calcification, thickness, and stiffness, clarifying their role in arterial remodeling processes.

## Introduction

Arterial thickness and calcification are a sequential process of arterial remodeling that occurs in response to chronic diseases, injuries, or aging, and leads to arterial stiffness^1,2^. Several mechanisms were involved in this sequential process, such as the following: (1) First, fibrosis and hyperplasia take place in arterial intima and media layers along with vascular smooth muscle cell (VSMC) migration and proliferation, which contributed to arterial thickness^1^; after that, (2) nucleation of calcium phosphate, extracellular matrix calcification, and increase arterial tone arise due to VSMC differentiation from the contractile to the secretory phenotype, which contributed to arterial calcification^1,3,4^, and then (3) loss of arterial wall elasticity occurs due to both previous processes that lead to arterial stiffness^2,5^. This sequential process may lead to various cardiovascular events, including myocardial infarction^6^, myocardial remodeling^7^, hypertension^8^, atherosclerosis^8^, stroke^6^, and chronic kidney disease^9^, which will eventually increase cardiovascular morbidity and mortality rates^10–12^. Moreover, this complex pathophysiology that started from arterial remodeling involves several proteins^13^. These proteins may become potential biomarkers and early prevention tools for cardiovascular events. Two of the most extensively studied proteins are Klotho and fibrovascular growth factor-23 (FGF-23), and both proteins were lately known to form the FGF-23/Klotho axis in arterial remodeling^14,15^.

FGFs compose a large family of proteins that affect development, organogenesis, and metabolism^16^. FGF-23 has been established as a novel biomarker involved in the development of cardiovascular diseases^17^. It is an endocrine hormone primarily released by osteocytes and plays a role in phosphate and vitamin D metabolism. FGF-23 regulates serum phosphate levels by downregulating sodium-phosphate cotransporter expression in the lumen of the proximal kidney tubules, further stimulating phosphaturia. FGF-23 also reduces the systemic levels of 1,25-dihydroxyvitamin D by inhibiting 1-α hydroxylase in the kidneys and stimulating the catabolic effects of 24-hydroxylase. Other actions include inhibiting the synthesis and secretion of parathyroid hormones^17,18^. The integrated effects of FGFs are mediated by their binding to FGF receptors (FGFRs), and recent studies have reported that this signaling requires Klotho proteins^18,19^.

Klotho proteins are a group of transmembrane proteins consisting of the following: α-Klotho, β-Klotho, and γ-Klotho protein^16^. They directly bind to multiple FGFRs to form Klotho-FGFR-complex, that are essentially required for the high-affinity binding of FGFs to their receptors^20^. Before the discovery of its homolog protein (β-Klotho), α-Klotho was also known as Klotho (which will be referred to hereinafter), and it serves as the obligate co-receptor for FGF-23. The expression of Klotho is downregulated by FGF-23^19^. Klotho is also present in the blood and urine in a soluble circulating form, which has been implicated in regulating endothelial integrity, permeability, and nitric oxide (NO) production^21^.

FGF-23 is expressed and secreted directly to the blood plasma by the bone, which then downregulates Klotho expression and followed by a reduction in Klotho soluble form generated by the proteolytic cleavage on the cell surface^22,23^. In an animal study, the deficiency of either FGF-23 or Klotho exhibited an impairment in the calcium phosphate metabolism and contributed to FGF-23/Klotho-mediated vascular calcification^11^, along with arterial thickness and stiffness^22^. However, the involvement of the FGF-23/Klotho axis in arterial calcification, thickness, or stiffness still needs to be elucidated whether or not it acts directly on human arteries and VSMCs. Although many studies focused on the connection between FGF-23 and Klotho on arterial calcification, thickness, and stiffness, but these studies are still controversial. Some studies showed significant correlation between FGF-23 or Klotho and arterial calcification/thickness/stiffness^24–26^, while some others did not^27–29^. Intriguingly, some other studies showed results different with theories, in which FGF-23 was inversely correlated with arterial pathologies^30^, but Klotho was positively correlated^31^. To the best of our knowledge, no meta-analyses have investigated the role of Klotho and FGF-23 in arterial remodeling, which prompted us to conduct a meta-analysis to establish their roles and prove their involvement in arterial calcification, thickness, and stiffness.

## Methods

This review was conducted according to Preferred Reporting Items for Systematic Reviews and Meta-Analyses (PRISMA 2020) guidelines^32^. The systematic review had been registered on PROSPERO (Registration no. CRD42021269744).

### Searching strategy

An electronic search was conducted on PubMed, Web of Science, EBSCO/CINAHL, Scopus, and Science Direct for articles up to December 2023. To limit the effect of publication bias, the gray literature was also searched for related articles, as database search alone is insufficiently rigorous. A mixture of Medical Subject Heading terms and free text were used to construct search terms using the following concepts: “Klotho,” “FGF-23,” “vascular calcification,” and “vascular stiffness.” The full search strategies are presented in Supplemental Table 1.

### Eligibility criteria

A PECO framework was employed to determine the study’s eligibility criteria, as shown below:

- **Patients:** Patients with arterial calcification, thickness, or stiffness. Arterial calcification was validated using a calcification score, arterial thickness was measured by the carotid intima–media thickness (CIMT), and arterial stiffness was assessed by the pulse wave velocity (PWV).
- **Exposure:** Klotho or FGF-23 levels.
- **Comparison:** None.
- **Outcomes:** Calcification score, CIMT, or PWV.

The inclusion criteria were as follows: (1) studies reporting the association of Klotho or FGF-23 level with arterial calcification, thickness, or stiffness; (2) measurement of arterial calcification, thickness, or stiffness used standard quantitative score; (3) English language; (4) observational study design; (5) human participants; and (6) reporting data in numerical values. The exclusion criteria were as follows: (1) review articles, cross-sectional studies, case reports, case series, and meta-analysis; (2) duplicated studies; (3) studies with incomplete or insufficient data; (4) abstract only or conference paper; and (5) insufficient data.

### Study selection and data extraction

Mendeley Desktop version 1.19.8 (Elsevier, Mendeley Ltd.) was used to remove duplicates and filter the studies. The extracted data were as follows: first author, publication year, country, sample size, age, study design, affected artery, diagnostic method, specified population, correlation coefficient (r), beta coefficient, odds ratio (OR) with 95% confidence intervals (CIs), and Klotho, or FGF-23 levels in groups with or without arterial calcification. Continuous data in the form of median and range were converted to mean and standard deviation by the method of Hozo et al.^33^. Beta coefficients were converted to ORs using exp(beta)^34^. In the case that data required for meta-analysis were not sufficient or not clearly reported in the paper, we contacted the authors.

Searching, study selection, and data extractions were independently conducted by two researchers (CDKW and CP) using a pre-specified form tabulated within the spreadsheet, and all data extraction tables were validated by two other researchers (HS and MYA). Quality assessments were performed independently by two researchers (BSW and APW) who used the Newcastle–Ottawa scale (NOS) for observational studies (cohort, case–control, and cross-sectional studies) to assess information bias, selection bias, and confounding. Studies with scores of 7–9, 4–6, and 0–3 were considered to have high, moderate, and low quality, respectively. Any conflicts or disagreements were resolved by discussion to achieve consensus.

### Statistical analysis

Each Spearman or Pearson correlation coefficient (r) was converted to a Z-value via Fisher’s transformation, which was approximately normally distributed^35,36^. The standard error of Z was calculated, and Z-values were converted via inverse Fisher’s transformation to generate r and 95% CI. The extracted ORs with 95% CIs were pooled to generate the overall adjusted ORs. Pooled standardized mean difference (SMD) and 95% CI were generated to analyze the difference in the Klotho or FGF-23 level between groups with and without arterial calcification.

The chi-squared test and I^2^ statistics was used to determine heterogeneity across studies. All analyses were pooled using a random-effects model. Sensitivity analysis was performed to guarantee the consistency of the results by omitting several factors that could influence the results (e.g., children and population aside from chronic kidney disease [CKD]). A one-leave-out sensitivity analysis was also performed by removing individual studies. If substantial heterogeneity occurred, subgroup analysis was employed to find the sources of heterogeneity. Publication bias was assessed visually through funnel plot asymmetry. In all analyses, a p-value of < 0.05 was considered statistically significant. Review Manager 5.4 (Cochrane Collaboration, London, UK) was used for this meta-analysis.

## Results

### Study characteristics

**Figure 1:**
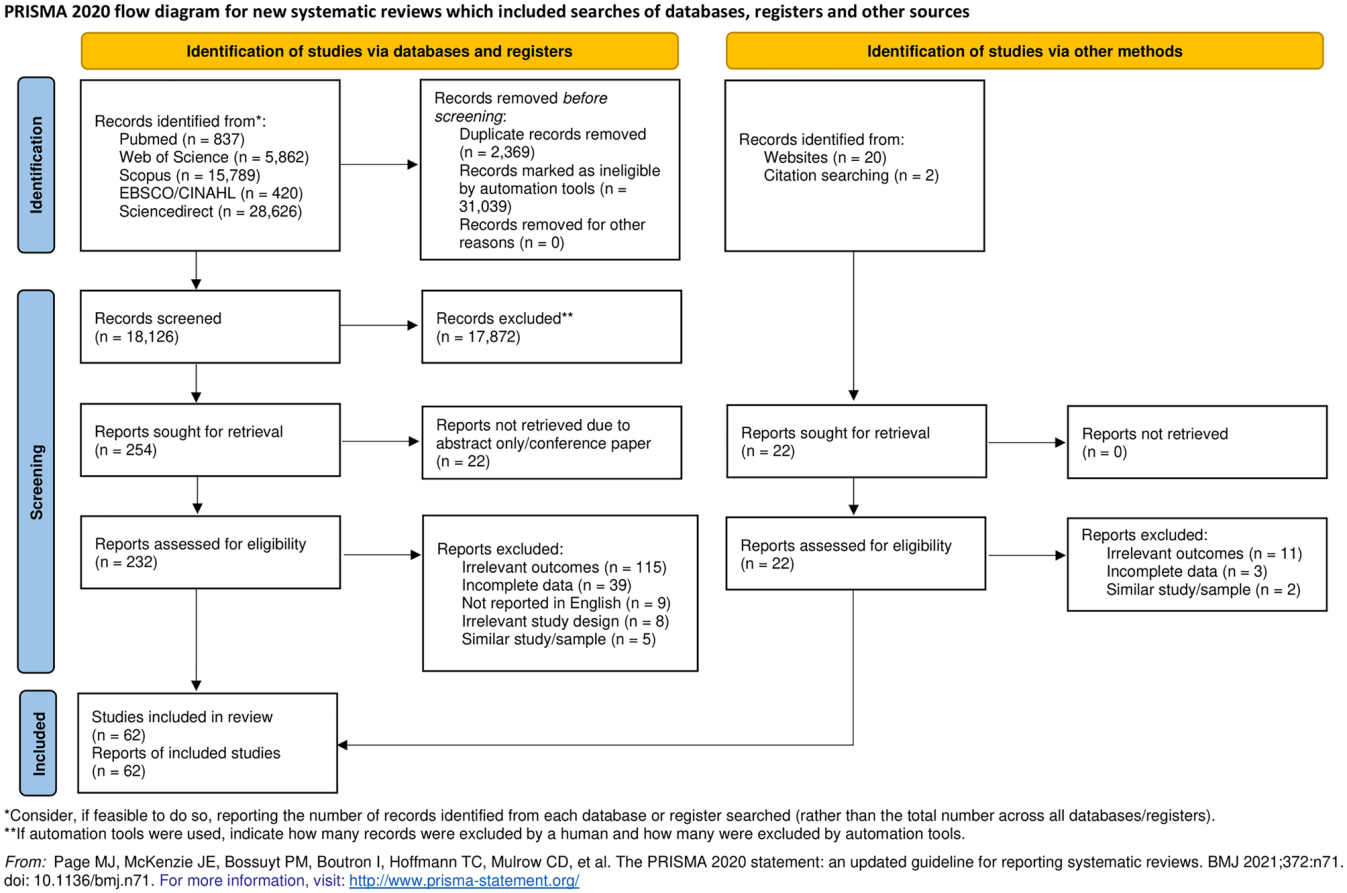
PRISMA flow diagram of the literature search.

The PRISMA flow diagram of the study selection process is shown in Fig. 1. In total, 51,534 eligible studies were documented from the searched electronic databases. Of the total articles, 31,039 were removed using automation filter tools from each database. Then, 2369 were removed for being duplicates, leaving 18,126 articles for further evaluation. Subsequently, 17,872 articles were excluded based on their titles and abstracts, whereas 254 papers were sought for retrieval. Another 22 articles were rejected for being conference abstracts and posters or having unavailable full-texts, leaving 232 articles for full-text article review. After full-text evaluation, 176 studies were further excluded because of irrelevant outcomes, incomplete data, non-English language, irrelevant study design, and similar study/sample. In addition, 22 extra records were identified from the website and reference list search. After judging the eligibility of the reports, 16 articles were excluded due to irrelevant outcomes, incomplete data, and similar study/sample. Ultimately, 62 articles were included in this meta-analysis.

Sixty-two publications, involving 27,459 participants, were eligible according to the inclusion and exclusion criteria. The primary features of the included studies are shown in Table 1. All included studies had an observational study design. In terms of continental regions, the majority of these 62 studies are from Asia (n = 29), including China (n = 12), followed by Europe (n = 16), America (n = 8), Africa (n = 8), and Australia (n = 1). Most studies have adult participants (aged ≥ 18 years), except for three studies involving children and adolescents. The majority of the participants had CKD (n = 46). Most of the studies had cross-sectional designs (n = 50), whereas the rest were cohort and case–control studies (n = 11 and n = 1, respectively). The measured arteries varied, with mostly focused on coronary, aorta, and carotid arteries. The arterial calcification score was measured either with computed tomography (CT) or X-ray imaging, except for the studies by Milovanova^37^ and Di Lullo^38^ which used echocardiography. On the contrary, CIMT, and PWV were mostly measured by ultrasonography. According to the sample for FGF-23/Klotho measurement, all studies used blood sample, either in the form of plasma or serum. Forty-eight studies used serum sample, while the rest used plasma. Most FGF-23/Klotho used enzyme-linked immunoassay (ELISA) method, except for one study which used Luminex and one study did not mention the method used. Four studies did not mention the ELISA kit used. Among ELISA kit used for FGF-23 analysis, Immunotopics were used the most (36%), followed by Kainos (30%), Elabscience (8%), and Millipore (6%). As for Klotho analysis ELISA kit, Immuno-Biological Laboratories were mostly used (50%), followed by Cusabio (27.78%).

**Table 1:** Characteristics of the included studies.

First author (year)	Country	Study design	Age (year)	Sample size	Characteristics of population	Affected artery	Outcomes	Correlation coefficient (r), p value	Odds ratio/Beta coefficient with confidence interval	Adjusted factors	Clinical measurement method for arterial calcification, thickness, or stiffness	Sample	Laboratory measurement method for FGF-23 or Klotho
Salam et al. (2021)^40^	UK, Europe	Cross sectional	62 ± 12	69	Advanced CKD	Lower leg artery	Correlation between FGF-23 and LLAC	0.397, p = 0.001	–	–	HRpQCT	Serum	Manual ELISA (Immutopics)
Yilmaz et al. (2015)^55^	Turkey, Asia	Cross sectional	32 ± 9	178	Renal transplants (stage 5 CKD)	Carotid	Correlation between FGF-23 and CIMT	0.36, p = 0.001	–	–	Doppler ultrasound	Serum	Second generation, two-site mAb ELISA (Kainos laboratories)
Craver et al. (2013)^68^	Spain, Europe	Cross sectional	CAC 75 ± 6 Non-CAC 61 ± 14	53 AAC, 57 no AAC	Stage 3 and 4 CKD	Abdominal aorta	Comparison of FGF-23 and Klotho levels between groups with/without arterial calcification	–	–	–	Kauppila index from lateral lumbar X rays	Serum	ELISA for C-Term (Immutopics for FGF-23 and Immuno-Biological Laboratories for Klotho)
Masai et al. (2013)^49^	Japan, Asia	Cross sectional	65.5 (55–72)	148	Suspected CAD	Coronary	Correlation between FGF-23 and Agatston score	0.169, p = 0.039	Beta 1.488 (0.448; 2.529)	Sex, age, CKD stage, carotid plaque, FEP	Agatston score from CT	Serum	Sandwich ELISA (Kainos laboratories)
Nitta et al. (2018)^30^	Japan, Asia	Cross sectional	Calcification: 71.8 ± 10.4 No calcification: 60.7 ± 14.0	101 calcification, 173 no calcification	MHD	Aortic arch	Correlation between FGF-23 and AoACS score	− 0.12, p = 0.0175	Beta − 0.120 (− 0.220 to − 0.021)	Age, gender, BMI, dialysis vintage, Kt/V, CaxP, Hb, albumin, sclerostin	Chest X rays	Serum	Sandwich ELISA (Kainos laboratories)
Nasrallah et al. (2010)^39^	Egypt, Africa	Cross sectional	50 ± 11.5	65	HD	Abdominal aorta	Correlation between FGF-23 and aortic calcification index	0.48, p = 0.0001	Beta 0.58 (0.001–0.002)	Age, dialysis vintage, diastolic blood pressure, parathormone, phosphate, triglycerides, cholesterol	CT of abdominal aorta	Serum	Two side ELISA (Immutopics)
Lee et al. (2016)^48^	Taiwan, Asia	Cross sectional	63.0 ± 10.1	227	MHD	Abdominal aorta	Correlation between FGF-23 and abdominal aortic calcification; comparison of FGF-23 levels between groups with/without arterial calcification	0.116, p = 0.019	Beta 1.940 (0. 614 to 3.267)	Age, BMI, diabetes, hypertension, vascular disease, calcium, calcium phosphate products, albumin, hsCRP, sclerostin, DKK-1,	Kauppila index from lateral lumbar X rays	Serum	ELISA (Immutopics)
Ibrahim et al. (2018)^59^	Egypt, Africa	Cross sectional	43 ± 14.2	128	HD	Unspecified	Correlation between FGF-23 and PWV	–	–	–	Doppler with 2D guidance and ECG trigger	Serum	Sandwich ELISA (Diagnostics Systems Laboratories)
Castelblanco et al. (2022)^88^	Spain, Europe	Cross sectional	52.2 ± 8.8 with plaque, 41.9 ± 10.3 no plaque	85 with plaque, 288 no plaque	Type 1 DM	Carotid	Comparison of FGF-23 and Klotho levels between groups with/without subclinical carotid atherosclerosis	–	–	–	CIMT from Carotid Ultrasound Imaging	Serum	ELISA (TECOmedical for FGF-23 and Cusabio Biotech for α-klotho)
Sandoval et al. (2015)^82^	Mexico, America	Cross sectional	50 ± 16 calcification, 41 ± 18 no calcification	22 calcification, 54 no/low calcification	Peritoneal dialysis	Unspecified	Comparison of FGF-23 levels between groups with/without arterial calcification	–	–	–	Adragao Score from plain radiographic films of pelvis and hands	Serum	Luminex/Magpix system
Fayed et al. (2019)^46^	Egypt, Africa	Cross sectional	43.68 ± 13.66	81	Recently starting HD	Abdominal aorta	Correlation between FGF-23 and abdominal aortic calcification	0.8, p < 0.001	–	–	Abdominal CT	Serum	ELISA (unspecified)
Pencak et al. (2013)^80^	Poland, Europe	Cross sectional	60 (57–63)	76 AAC + CAC, 10 no calcification	HD	Abdominal aorta and coronary	Comparison of FGF-23 levels between groups with/without arterial calcification	–	–	–	Agatston score from CT	Plasma	ELISA (Immutopics)
Srivaths et al. (2014)^83^	US, America	Cross sectional	19.7 ± 1.5 CAC, 16.2 ± 3.2 no CAC	6 CAC, 10 no CAC	Pediatric HD	Coronary	Comparison of FGF-23 levels between groups with/without arterial calcification	–	–	–	Agatston score from CT	Serum	ELISA (Immutopics)
Milovanova et al. (2022)^37^	Russia, Europe	Cross sectional	20–65	130	CKD	Coronary	Correlation between Klotho and abdominal CCS/ PWV	Klotho—CCS: − 0.581, p < 0.01; Klotho—PWV: − 0.66, p < 0.001	–	–	CCS Echocardiography	Serum	ELISA (Merck Millipore for FGF-23 and Immuno-Biological Laboratories for Klotho)
Yu et al. (2018)^67^	China, Asia	Cross sectional	63.43 ± 12.76	330	HD	Carotid	Correlation between Klotho and CIMT	− 0.183, p = 0.001	–	–	Doppler ultrasound	Serum	ELISA (Immuno-Biological Laboratories)
Cianciolo et al. (2010)^45^	Italy, Europe	Cross sectional	Males 62.5 ± 13.5, females 60.5 ± 11.5	253	ESRD	Coronary	Correlation between FGF-23 and CAC, comparison of FGF-23 levels between groups with/without arterial calcification	− 0.23, p = 0.02	–	–	Agatston score from CT	Plasma	ELISA (Immutopics)
Muzasti et al. (2021)^50^	Indonesia, Asia	Cross sectional	57 (25–78)	75	HD	Abdominal aorta	Correlation between FGF-23 and AAC	0.543, p < 0.001	–	–	Lateral lumbar X rays	Serum	Two-site ELISA (unspecified)
Baralic et al. (2019)^69^	Serbia, Europe	Cross sectional	54 ± 13	56	HD	Iliac, femoral, radial and digital arteries	Association between FGF-23 and vascular score	–	OR 1.006 (0.992 to 1.012)	–	Adragao score from radiographic films of pelvis and hands	Serum	ELISA (Cusabio)
He et al. (2017)^90^	China, Asia	Cross sectional	LEAD: 62 (59–68) No LEAD: 50 (41–58)	201 LEAD, 200 no LEAD	Type 2 DM	Lower extremities arteries	Comparison of FGF-23 levels between groups with/without lower extremities arterial thickness	–	–	–	Doppler ultrasound	Serum	ELISA (Kainos)
Ford et al. (2011)^58^	UK, Europe	Cross sectional	69 ± 11	200	Stage 3 and 4 CKD	Aorta	Correlation between FGF-23 and PWV	0.262, p < 0.001	–	–	Complior	Serum	Sandwich ELISA (Immutopics)
Mudi et al. (2019)^53^	South Africa, Africa	Cross sectional	10.8 ± 3.5	72	CKD children	Carotid	Correlation between FGF-23 and CIMT	0.222, p = 0.061	–	–	Doppler ultrasound	Plasma	ELISA (Merck Millipore)
Ortiz et al. (2020)^54^	Spain, Europe	Cross sectional	59.5 ± 0.3	939	Non-CKD CHD	Carotid	Correlation between FGF-23 and CIMT	0.16, p < 0.001	–	–	Doppler ultrasound	Serum	ELISA (Kainos)
Koga et al. (2021)^61^	Japan, Asia	Cross sectional	68 ± 9	75	Stable CHD	Coronary	Correlation between Klotho and CAC	− 0.31, p = 0.007	–	–	Intravascular ultrasound	Serum	ELISA (Immuno-Biological Laboratories)
Balci et al. (2010)^87^	Turkey, Asia	Cross sectional	55.5 ± 13	128	MHD	Carotid	Comparison of FGF-23 levels between groups with/without arterial thickness	–	–	–	Doppler ultrasound	Plasma	ELISA for C-Term (Immutopics)
Bundy et al. (2018)^70^	US, America	Cross sectional	CAC 60.6 ± 9.3, no CAC 50.9 ± 12.2	689 CAC, 434 no CAC	Mild to moderate CKD	Coronary	Comparison of FGF-23 levels between groups with/without CAC	–	OR 1.32 (1.05 to 1.67)	Age, sex, race/ethnicity, clinical site, follow-up time between CT scans, total cholesterol, HDL cholesterol, systolic BP, use of antihypertensive medications, diabetes, current smoking, history of CVD, use of statin medications, and physical activity	Agatston score from CT	Serum	ELISA for C-Term (Immutopics)
Cai et al. (2015)^60^	China, Asia	Cross sectional	58.18 ± 13.72	129	MHD	Abdominal aorta	Correlation between Klotho and AAC	− 0.214, p = 0.015	–	Age, gender, smoking	Kauppila index from abdominal aorta plain roentgenography	Serum	Sandwich ELISA (Immuno-Biological Laboratories)
Keles et al. (2016)^65^	Turkey, Asia	Cross sectional	33 (29–40)	80	Type 1 DM	Carotid	Correlation between Klotho and CIMT	− 0.594, p = 0.001	–	–	Ultrasound	Serum	ELISA (Cusabio Biotech)
Chen et al. (2013)^24^	China, Asia	Cross sectional	55.1 ± 14.9	120	MHD	Abdominal aorta	Correlation between FGF-23 and AAC	0.371, p < 0.001	OR 2.366 (1.304–4.291)	PTH	Lateral lumbar X rays	Plasma	ELISA for C-Term (Immutopics)
Coban et al. (2018)^51^	Turkey, Asia	Cross sectional	47.8 ± 13.9	86	Autosomal Dominant Polycystic Kidney Disease	Carotid	Correlation between FGF-23 and PWV/CIMT	FGF-23—PWV: 0.337, p = 0.002; FGF-23—CIMT: 0.298, p = 0.005	–	–	Ultrasound	Serum	ELISA (Elabscience)
Jasani et al. (2018)^77^	India, Asia	Cross sectional	48.5 ± 12.8	60 CAC, 40 no CAC	HD	Coronary	Comparison of FGF-23 levels between groups with/without CAC	–	–	–	Agatston score from CT	Serum	ELISA (Immutopics)
Jeong et al. (2013)^63^	South Korea, Asia	Cross sectional	47.2 ± 8.1 with subclinical carotid atherosclerosis, 38.5 ± 8.1 without subclinical carotid atherosclerosis	140	HIV	Carotid	Correlation between Klotho and CIMT, comparison of Klotho levels between groups with/without subclinical carotid atherosclerosis	− 0.258, p = 0.004	OR 0.006 (0.000–0.677)	Age, HIV, total cholesterol, gender, stavudine use	Ultrasound	Plasma	Sandwich ELISA (Immuno-Biological Laboratories)
Villodres et al. (2019)^84^	Spain, Europe	Cross sectional	58 ± 8	45 AAC, 35 no AAC	Stage 3 CKD	Abdominal aorta	Comparison of FGF-23 and Klotho levels between groups with/without AAC	–	–	–	Agatston score from abdominal CT	Serum	Direct sandwich ELISA (Kainos for FGF-23 and Cusabio for Klotho)
Keles et al. (2015)^64^	Turkey, Asia	Cross sectional	32 (27–38)	50	Healthy adults	Carotid	Correlation between Klotho and CIMT	− 0.522, p < 0.001	–	–	Ultrasound	Serum	ELISA (Cusabio Biotech)
Figurek et al. (2018)^52^	Bosnia, Europe	Cross sectional	62.86 ± 11.43	87	CKD	Carotid	Correlation between FGF-23 and CIMT	0.12, p > 0.05	–	–	Ultrasound	Serum	ELISA (Elabscience)
Morita et al. (2015)^31^	Japan, Asia	Cross sectional	Men 67 ± 11.6, women 68.5 ± 11.5	157	Subjects diagnosed or suspected with CAD	Coronary and aortic valve	Correlation between FGF-23/ Klotho and Agatston score	FGF-23—Agatston score: 0.244, p = 0.035; Klotho—Agatston score: − 0.058, p = 0.621	OR FGF-23—CAC 2.39 (0.73 to 7.88); OR FGF-23—AVC 1.73 (0.57 to 5.2)	Age, CKD stage, hypertension, statin, diuretics, cCa, P, PTH, and vitamin D	Agatston score from CT	Serum	ELISA (Kainos for FGF-23 and Immunobiological Laboratories for Klotho)
Nakayama et al. (2013)^72^	Japan, Asia	Cross sectional	71.9 ± 9.4 CAC, 62.7 ± 12.3 no CAC	54 CAC, 34 no CAC	Non-HD CKD	Carotid	Comparison of FGF-23 levels between groups with/without carotid calcification	–	OR 1.75 (1.01 to 3.04)	Age, sex, hypertension, DM, smoking, dyslipidemia, BMI, proteinuria, CRP, Hb, P, Ca-P, eGFR	Agatston score from CT	Serum	ELISA (Kainos)
Schoppet et al. (2012)^73^	France, Europe	Cross sectional	72 ± 7	780	Healthy adults	Abdominal aorta	Association between FGF-23 and abdominal aortic calcification	–	OR 1.25 (1.03 to 1.53)	Age, weight, ischemic heart disease, hypertension, diabetes mellitus, and vitamin D	Dual-energy X-ray absorptiometry	Serum	ELISA (Immutopics)
Singh et al. (2022)^29^	India, Asia	Cross sectional	10.1	59	CKD children	Carotid	Correlation between FGF-23 and CIMT/PWV	FGF-23—CIMT: − 0.195, NS; FGF-23—PWV: 0.183, NS	–	–	Ultrasound	Serum	ELISA (Elabscience)
Zeng et al. (2015)^56^	China, Asia	Cross sectional	56.19 ± 14.1	87	PD	Carotid	Correlation between FGF-23 and CIMT	0.628, p < 0.0001	–	–	Ultrasound	Plasma	ELISA (unspecified)
Zhang et al. (2015)^43^	China, Asia	Cross sectional	56.77 ± 10.41	200	CKD stage 3–5	Coronary	Correlation between FGF-23 and CAC	0.177, p = 0.034	–	–	Agatston score from CT	Serum	Two-site ELISA (Kainos)
Zayed et al. (2015)^42^	Egypt, Africa	Cross sectional	52	80	MHD	Coronary	Correlation between FGF-23 and CAC	0.7, p = 0.001	–	–	Agatston score from CT	Serum	Two-site ELISA (Immutopics)
El Baz et al. (2017)^25^	Egypt, Africa	Cross sectional	55.8 ± 9.4 calcification, 52.5 ± 9.1 no calcification	60	ESRD	Coronary and abdominal aorta	Correlation between FGF-23 and CAC/AAC, comparison of FGF-23 levels between groups with/without arterial calcification	FGF—CAC: 0.682, p < 0.001; FGF—AAC: 0.606, p < 0.001	–	–	Agatston score from CT	Plasma	Not specified
Zaki et al. (2018)^41^	Egypt, Africa	Cross sectional	18–70	90	HD	Abdominal aorta	Correlation between FGF-23 and aortic calcification	0.964, p < 0.001	–	–	CT	Serum	ELISA (unspecified)
Tarigan et al. (2019)^66^	Indonesia, Asia	Cross sectional	54.21 ± 10.86	70	MHD	Carotid	Correlation between Klotho and CIMT	− 0.368. p = 0.002	–	–	Ultrasound	Plasma	ELISA (Bio Vendor)
Gutierrez et al. (2009)^71^	US, America	Cross sectional	≧30	162	Predialysis CKD	Coronary	Association between FGF-23 and CAC	–	OR 1.2 (0.8 to 1.7)	–	Agatston score from CT	Serum	Two-site ELISA (Immutopics)
Lin et al. (2022)^62^	China, Asia	Cross sectional	52.15 ± 8.8	98	MHD	Abdominal aorta, iliac, femoral, radial, and digital arteries	Correlation between Klotho and arterial calcification	− 0.72, p < 0.0001	–	–	Plain radiographic images	Serum	ELISA (R&D Systems)
Turan et al. (2016)^44^	Turkey, Asia	Cross sectional	58.7 ± 14.2	229	HD	Coronary artery	Correlation between FGF-23 and arterial calcification	r = 0.218, p = 0.001	–	–	Agatston score from CT	Plasma	ELISA (Immutopics)
Zhu (2023)^75^	China, Asia	Cross sectional	55.8 ± 14.9	58 CAC, 70 no CAC	Non-HD CKD	Coronary artery	Comparison of FGF-23 between groups with/without CAC, association between FGF-23 and CAC	–	OR 0.896 (0.257–3.118)	Age, DM, vitamin D, C1q/tumor necrosis factor-related protein-3	Agatston score from CT	Serum	ELISA (Elabscience)
Cancela (2012)^85^	Brazil, South America	Cross sectional	58.1 ± 9.3	169 CAC, 121 no CAC	Suspected CAD with normal renal function	Coronary artery	Comparison of FGF-23 between groups with/without CAC	–	–	–	Agatston score from CT	Serum	ELISA (Kainos)
Zamparini (2018)^57^	South Africa, Africa	Cross sectional	29.8 ± 13.6 patient group, 28.8 ± 13.01 control groups	30 patient group, 30 control group	Familial hypercholesterolemia	Carotid artery	Correlation between FGF-23 and CIMT	r = − 0.2656, p > 0.05	–	–	Ultrasound	Serum	ELISA (Kainos)
Guo et al. (2021)^89^	China, Asia	Case control	68.85 ± 7.45 with plaque, 46.62 ± 5.51 no plaque	40 with plaque, 33 no plaque	CAPD	Carotid	Comparison of FGF-23 and Klotho levels between groups with/without carotid artery thickness	–	–	–	CIMT from Carotid Ultrasound Imaging	Serum	ELISA (Millipore)
Ge et al. (2022)^76^	China, Asia	Cohort	53.61 ± 11.72 calcification, 39.76 ± 10.30 no calcification	38 CAC, 25 no CAC	MHD	Coronary	Comparison of FGF-23 levels between groups with/without arterial calcification	–	–	–	Agatston score from Coronary CT	Plasma	ELISA for C-Term (Immutopics)
Petrauskiene et al. (2018)^81^	Lithuania, Europe	Cohort	65.2 ± 14.4 calcification, 53.97 ± 16.2 no calcification	50 calcification, 31 no calcification	HD	Unspecified	Comparison of FGF-23 levels between groups with/without arterial calcification	–	–	–	Adragao Score from plain radiographic films of pelvis and hands	Serum	ELISA (Sunlong Biotech)
Bortnick et al. (2019)^86^	US, America	Cohort	70 ± 8 AVC, 61 ± 10 no AVC	913 AVC, 5899 no AVC	Multi ethnic non-CVD	Aortic valve	Comparison of FGF-23 levels between groups with/without arterial calcification	–	–	–	Agatston score from Coronary CT	Serum	ELISA (Kainos)
Di Lullo et al. (2015)^38^	Italy, Europe	Cohort	51 (46–56)	100	Mild to moderate CKD	Aortic valve	Correlation between Klotho and AVC	− 0.208, p = 0.04	Coef Beta 0.116 (0.048 to 0.183)	PTH	Echocardiography	Serum	ELISA for C-Term (Biocompare Laboratories)
Krishnasamy et al. (2017)^47^	Australia, Australia	Cohort	≧18	40	Advanced CKD	Abdominal aorta	Correlation between FGF-23/ Klotho and AAC	FGF-23—AAC: 0.5, p < 0.001; Klotho—AAC: − 0.36, p = 0.002	OR FGF-23 and AAC 2.61 (1.41 to 6.98)	Age, diabetes, hypertension, eGFR, corrected calcium, phosphate, and vitamin D	Lateral lumbar X rays	Serum	ELISA (Kainos for FGF-23 and Immuno-Biological Laboratories for Klotho
Kurnatowska et al. (2011)^78^	Poland, Europe	Cohort	62.3 ± 10.9 CAC, 53.1 ± 1.0 no CAC	33 CAC, 14 no CAC	HD	Coronary	Comparison of FGF-23 levels between groups with/without CAC	–	–	–	Agatston score from CT	Plasma	ELISA (ALPCO Diagnostics)
Linefsky et al. (2014)^79^	US, America	Cohort	70.49 ± 8.1 AVC, 60.86 ± 9.9 no AVC	913 AVC, 5899 no AVC	Multi ethnic study	Aortic valve	Comparison of FGF-23 levels between groups with/without AVC	–	–	–	Agatston score from CT	Serum	Sandwich ELISA (Kainos)
Zhu et al. (2019)^74^	China, Asia	Cohort	60.19 ± 12.15 no to minor calcification, 55.02 ± 14.20 moderate to severe calcification	61 no to minor calcification, 53 moderate to severe calcification	HD	Abdominal aorta	Comparison of FGF-23 levels between groups with/without abdominal aortic calcification	–	OR 2.83 (1.01 to 7.94)	Age, dialysis vintage, smoking, logFGF23, Kt/v, hsCRP, HDL, iPTH, and calcitriol use	Kauppila index from lateral abdominal radiographs	Serum	ELISA (Kainos)
Zheng et al. (2018)^26^	China, Asia	Cohort	61.91 ± 15.39	128	MHD	Coronary	Correlation between Klotho and CAC	− 0.667, p = 0.001	–	–	Agatston score from CT	Serum	ELISA (R&D)
Buiten et al. (2014)^103^	Netherlands, Europe	Cohort	67 ± 7	127	HD	Coronary and abdominal aorta	Association between Klotho and AAC/CAC	–	Beta Klotho AAC: 0.58 (− 0.07 to 1.22); CAC: 0.08 (− 0.19 to 0.36)	Age, gender, dialysis vintage, dialysis type, and residual renal function	Agatston score from CT for CAC, lateral abdominal X rays for AAC	Plasma	ELISA (Immuno-Biological Laboratories)
Scialla et al. (2013)^27^	US, America	Cohort	57 ± 12	3939	Mild to moderate CKD	Coronary	Association between FGF-23 and CAC/TAC	–	OR CAC 1.02 (0.90 to 1.16); TAC 1.06 (0.93 to 1.21)	Age, sex, race, ethnicity, eGFR, ln-transformed urine albumin-to-creatinine ratio, prior cardiovascular, disease, DM, hypertension, hypercholesterolemia, smoking, BMI, corrected serum calcium, PTH, and clinical center	Agatston score from CT	Plasma	Second generation C-terminal assay (Immutopics)

*AAC* abdominal aortic calcification, *AoACS* aortic arch calcification score, *AVC* aortic valve calcification, *BMI* body mass index, *Ca* calcium, *CAC* coronary artery calcification, *CAD* coronary artery disease, *CAPD* continuous ambulatory peritoneal dialysis, *CCS* cardiac calcification score, *CHD* coronary heart disease, *CKD* chronic kidney disease, *CT* computed tomography, *CVD* cardiovascular disease, *DKK-1* Dickkopf-1, *DM* diabetes mellitus, *eGFR* estimated glomerular filtration rate, *ELISA* enzyme-linked immunoassay, *ESRD* end stage renal disease, *FEP* fractional excretion of phosphate, *Hb* hemoglobin, *HRpQCT* High resolution peripheral quantitative computed tomography, *hsCRP* high-sensitivity C-reactive protein, *LEAD* lower extremity atherosclerotic disease, *LLAC* lower leg arterial calcification, *MHD* maintenance hemodialysis, *NS* not significant, *P* phosphate, *PD* peritoneal dialysis, *PTH* parathyroid hormone, *PWV* pulse wave velocity.

Among the studies, sixteen^24,25,30,31,39–50^ reported correlations between the FGF-23 level and the calcification score, eight^29,51–57^ reported correlations between the FGF-23 level and the CIMT, and five^29,47,51,58,59^ reported correlations between the FGF-23 level and the PWV. Regarding Klotho, eight studies^26,31,37,38,47,60–62^ reported correlations between the Klotho level and the calcification score and five studies^63–67^ reported correlations between the Klotho level and the CIMT. For the regression analysis, seven studies^30,38,39,47–49,68^ reported an association between the FGF-23 level and arterial calcification in the linear regression model and ten studies^24,27,31,69–75^ reported an association between the FGF-23 level and arterial calcification in the logistic regression model. For continuous data, twenty studies^25,30,45,48,68,70,72,74–86^ reported a difference in FGF-23 levels between the group with and without arterial calcification, four^87–90^ reported a difference in FGF-23 levels between groups with arterial thickness, and three^63,88,89^ reported a difference in Klotho levels between groups with arterial thickness.

### Quality assessment

The quality of the 62 included studies was assessed using the NOS, which was suitable for each study design. Among those studies, only one study^46^ was considered to have low quality, 33 as moderate quality, and 28 as high quality. The quality assessment of each study using the NOS critical appraisal checklist is listed in Tables S3–S5.

### Correlations between FGF-23 levels and arterial calcification

**Figure 2:**
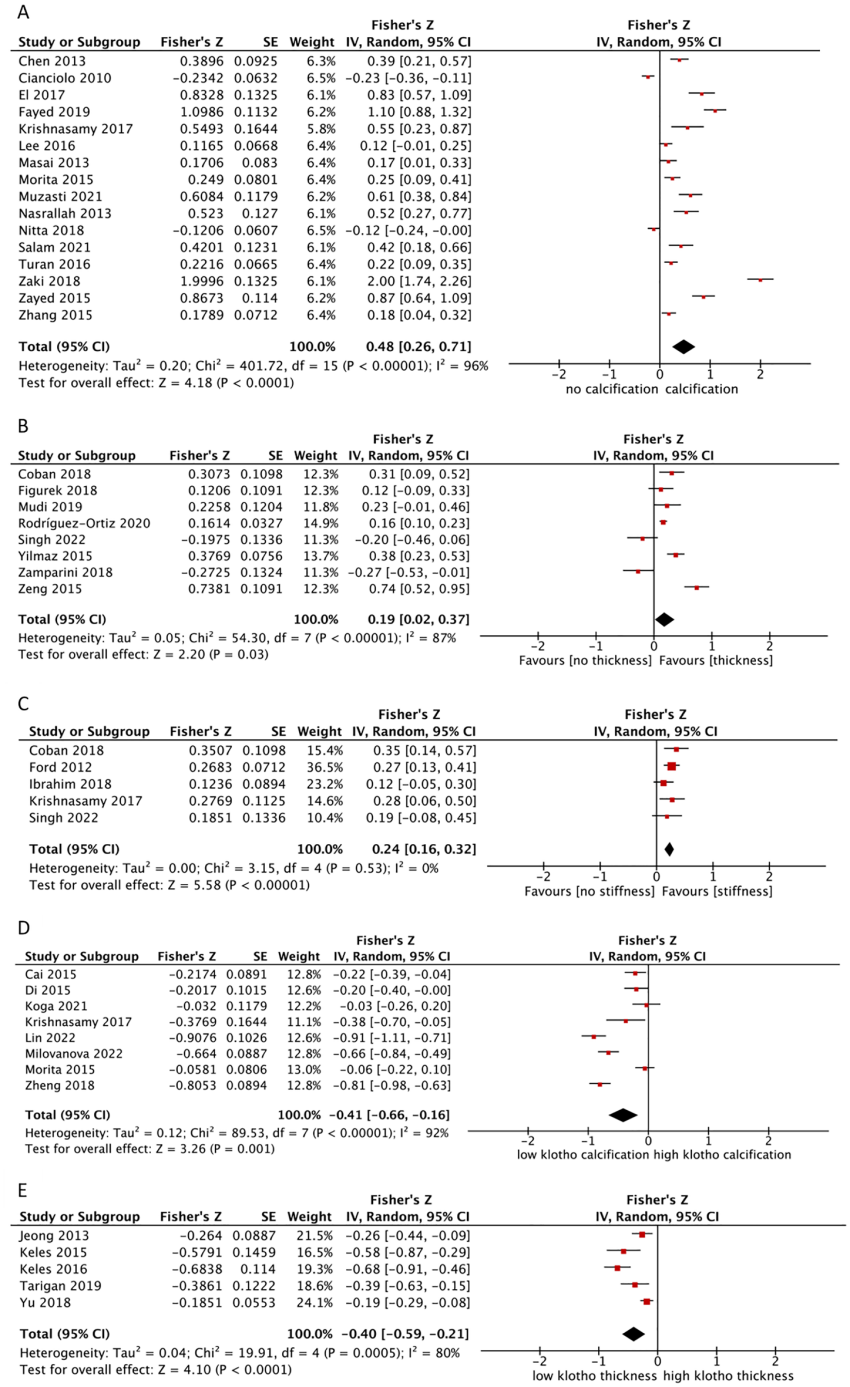
Forest plot of the pooled r for the correlation between: (**A**) FGF-23 level and arterial calcification; (**B**) FGF-23 level and CIMT; (**C**) FGF-23 level and PWV; (**D**) Klotho level and arterial calcification; (**E**) Klotho level and CIMT. All analyses are pooled using a random-effects model.

In sixteen studies, a moderate correlation was found between the FGF-23 level and arterial calcification [pooled r = 0.446 (0.254–0.611), p < 0.0001] (Fig. 2A). After sensitivity analysis by including CKD-only population (all in severe stage), cross-sectional study design, diagnosis of arterial calcification by CT, and high-quality studies, the results did not change much. However, when we perform sensitivity analysis for suspected coronary artery disease (CAD) only and diagnosis of arterial calcification by X-rays, the pooled correlations were given by r = 0.207 (CI = 0.1–0.31, n = 2, p-value 0.0002) and r = 0.282 (CI = 0.02–0.508, n = 5, p-value = 0.03), respectively. The correlation remains statistically significant at the 5% significance level, but the pooled r is lower than the correlation in the previous pooled analysis. In addition, we did not conduct sensitivity analysis for adults only since all studies regarding correlations between FGF-23 levels and arterial calcification score took adults patients only.

### Correlation between the FGF-23 level and the CIMT or PWV

Eight studies reported a weak correlation between the FGF-23 level and CIMT. In the pooled analysis, the FGF-23 level positively correlated with CIMT [pooled r = 0.188 (0.02–0.354), p = 0.03] (Fig. 2B). Analysis of the correlation between the FGF-23 level and PWV also showed a significant positive correlation [pooled r = 0.235 (0.159–0.310), p < 0.00001] (Fig. 2C), in which all included studies involved CKD patients. The sensitivity analysis excluded children and included studies with severe CKD-only; however, the results were still consistent.

### Correlation between the Klotho level and arterial calcification or CIMT

In contrast to FGF-23, an inverse correlation was found between the Klotho level and arterial calcification [pooled r = − 0.388 (− 0.578 to − 0.159), p = 0.001] (Fig. 2D). However, after including high-quality studies in the analysis, the pooled r changed [− 0.159 (− 0.264 to − 0.05), p = 0.005] along with reduced heterogeneity (47%). A significant negative correlation was also found between the Klotho level and CIMT [pooled r = − 0.38 (− 0.53 to − 0.207), p < 0.00001] (Fig. 2E). After including studies with the CKD-only population and high-quality studies only, the results remained stable. A meta-analysis for the correlation between the Klotho level and PWV was not performed as there was not enough number of studies that reported the correlation.

### Association between the FGF-23 and arterial calcification

**Figure 3:**
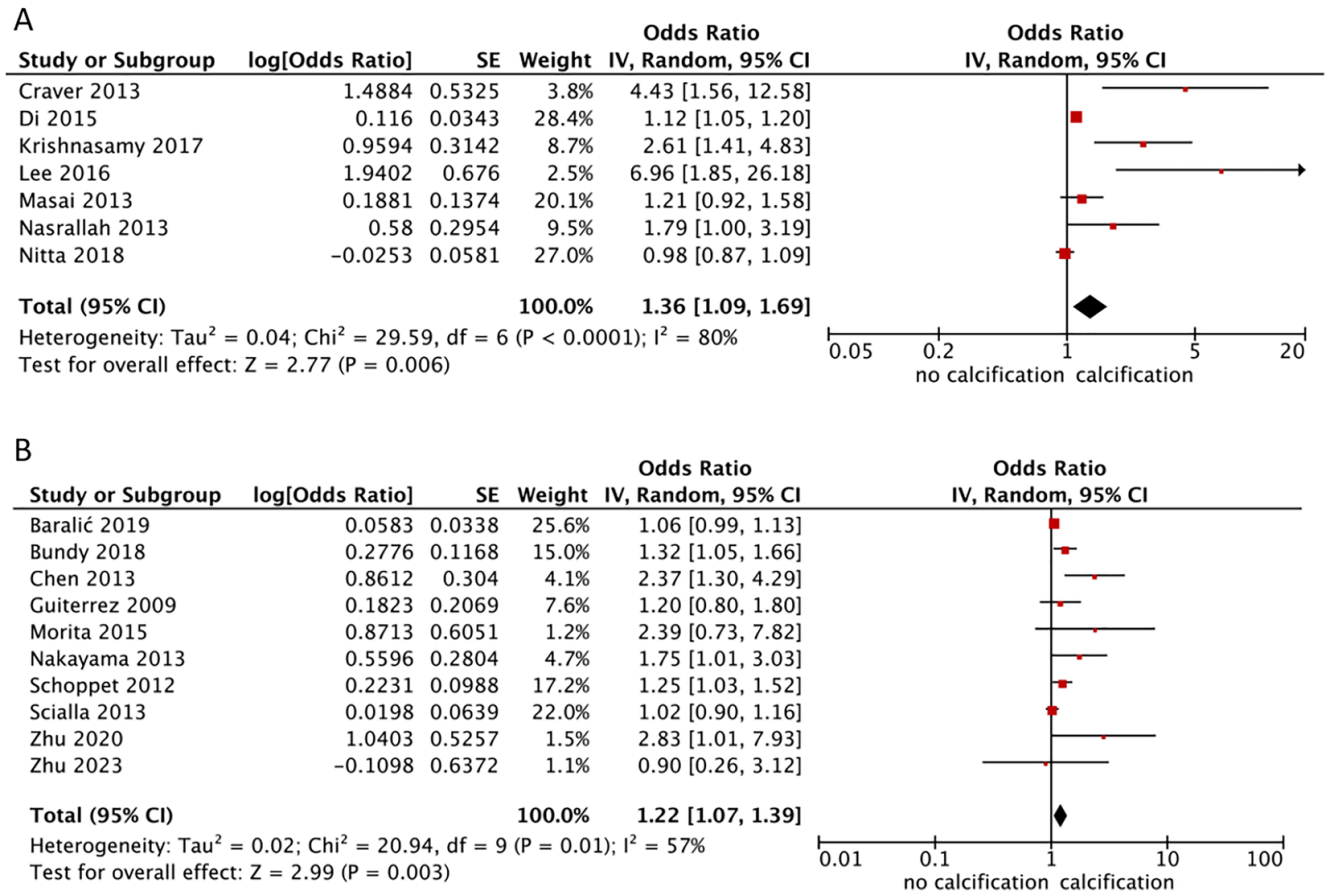
Forest plot of the pooled OR for the association between: (**A**) FGF-23 level and arterial calcification in linear regression model and (**B**) FGF-23 level and arterial calcification in logistic regression model. All analyses are pooled using a random-effects model.

Seven studies have reported ORs/beta and CIs for the association between the FGF-23 level and arterial calcification generated using multivariate linear regression, and nine reported using a logistic regression model. The extracted effectors in the original studies were generated after adjustment for important confounders including age, sex, estimated glomerular filtration rate, minerals (Ca/P), smoking, dialysis vintage, albumin, sclerostin, parathyroid hormone, vitamin D, and comorbidities. The pooled aOR was 1.36 (1.09–1.69) (p = 0.006) (Fig. 3A). For the logistic regression for the association between the FGF-23 level and arterial calcification, the pooled aOR was 1.22 (1.07–1.39) (p = 0.003) (Fig. 3B). In the sensitivity analysis that included CKD-only population and high-quality studies only, the results remained stable for both linear and logistic regression models. We did not perform pooled aOR analysis for Klotho due to limited data and varied concept of analysis between studies.

### FGF-23 level in groups with arterial calcification and arterial thickness

**Figure 4:**
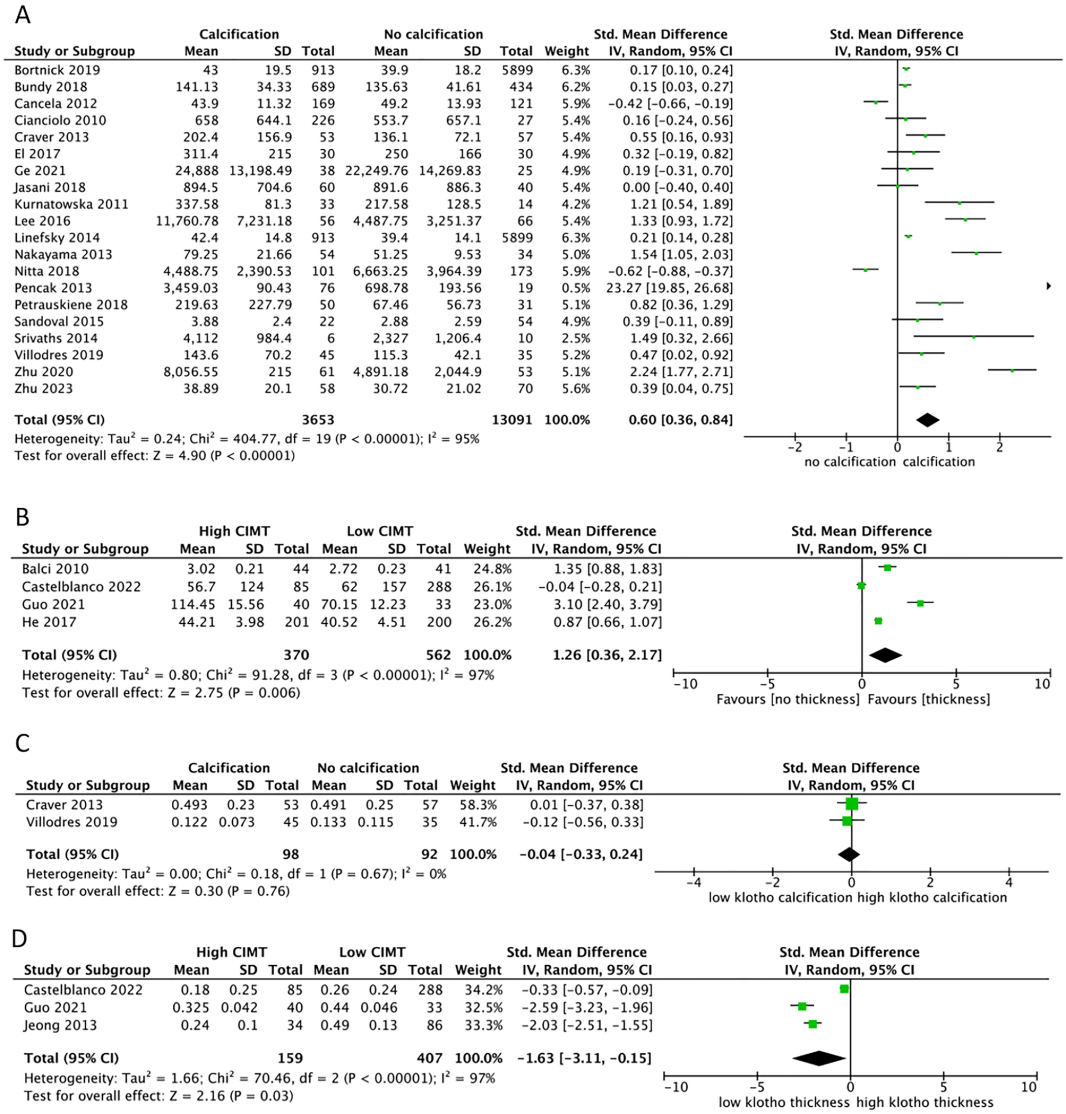
Forest plot of the pooled SMD for: (**A**) FGF-23 level in calcification/no calcification groups; (**B**) FGF-23 level in high CIMT/low CIMT groups; (**C**) Klotho level in calcification/no calcification groups; (**D**) Klotho level in high CIMT/low CIMT group. All analyses are pooled using a random-effects model.

An analysis of pooled SMD was also performed by comparing FGF-23 and Klotho levels between groups with and without arterial calcification. The group with arterial calcification had significantly higher FGF-23 levels than the group without arterial calcification [pooled SMD = 0.6 (0.36–0.84), p < 0.00001] (Fig. 4A). After conducting sensitivity analysis by including CKD-only population, measurement of calcification by the Agatston score or Kauppila index only, coronary artery only, and high-quality studies only, the results remained consistent. In subgroup analysis, the results of studies involving mild to moderate CKD only and severe CKD only also yielded consistent results. By comparing FGF-23 level difference between the groups with and without arterial thickness, the FGF-23 level was also significantly higher in the group with arterial thickness [pooled SMD = 1.26 (0.36–2.17), p = 0.006] (Fig. 4B).

### Klotho level in groups with arterial calcification and arterial thickness

Two studies^68,84^ have reported Klotho level differences between the groups with and without arterial calcification. However, a significant difference in Klotho levels was not found between the two groups [pooled SMD = − 0.04 (− 0.33 to 0.24), p = 0.76] (Fig. 4C). Meanwhile, a significantly lower Klotho level was found in the group with arterial thickness [pooled SMD = − 1.63 (− 3.11 to − 0.15), p = 0.03] (Fig. 4D). Sensitivity analysis revealed that the study by Castelblanco et al.^88^ had a significant effect on heterogeneity. After removing this study, the pooled SMD was − 2.27 (− 2.82 to − 1.72) (p < 0.00001), and the I^2^ was 49%. All analyses are summarized along with their sensitivity analyses in Table 2 for FGF-23 and Table 3 for Klotho.

**Table 2:** Summary of meta-analysis of FGF-23 with each sensitivity analysis.

Analysis and subgroup analysis	Effect measure	Pooled effect (95% CI)	I^2^ (%)	n	P value	Analysis model
Correlation between FGF-23 level and calcification score	Pooled r	0.446 (0.254–0.611)	96	16	< 0.0001	RE
CKD only (all severe)		0.478 (0.254–0.658)	97	14	< 0.0001	RE
Suspected CAD only		0.207 (0.1–0.31)	0	2	0.0002	RE
Cross-sectional only		0.446 (0.235–0.611)	96	15	< 0.0001	RE
Diagnosis by CT		0.515 (0.245–0.706)	97	11	0.0004	RE
Diagnosis by X ray		0.282 (0.02–0.508)	92	5	0.03	RE
High quality studies only		0.254 (0.09–0.405	86	7	0.003	RE
Correlation between FGF-23 level and CIMT	Pooled r	0.188 (0.02–0.354)	87	8	0.03	RE
Excludes children		0.245 (0.05–0.414)	89	6	0.02	RE
CKD only		0.264 (0.05–0.454)	85	6	0.02	RE
Severe CKD only		0.5 (0.197–0.716)	86	2	0.002	RE
Correlation between FGF-23 level and PWV (all subjects were CKD)	Pooled r	0.235 (0.159–0.310)	0	5	< 0.00001	RE
Excludes children		0.245 (0.159–0.327)	0	4	< 0.00001	RE
Severe CKD only		0.235 (0.1–0.363)	29	3	0.0007	RE
Linear regression for association between FGF-23 level and arterial calcification	Pooled aOR	1.36 (1.09–1.69)	80	7	0.006	RE
CKD only		1.44 (1.11–1.86)	83	6	0.006	RE
High quality studies only		1.58 (1.06–2.37)	82	5	0.03	RE
Logistic regression for association between FGF-23 level and arterial calcification	Pooled aOR	1.22 (1.07–1.39)	57	10	0.003	RE
CKD only		1.21 (1.04–1.41)	60	8	0.01	RE
High quality studies only		1.23 (1.07–1.42)	61	9	0.004	RE
FGF-23 levels difference between groups with and without arterial calcification	Pooled SMD	0.6 (0.36–0.84)	95	20	< 0.00001	RE
CKD only		0.95 (0.5–1.4)	96	17	< 0.0001	RE
Mild to moderate CKD only		0.33 (0.04–0.62)	62	3	0.02	RE
Severe CKD only		1.4 (0.64–2.17)	97	12	0.0003	RE
Based on Agatston score		0.47 (0.22–0.72)	95	14	0.0002	RE
Based on Kauppila index		1.23 (0.52–1.93)	91	4	0.0006	RE
Coronary only		0.87 (0.29–1.44)	96	10	0.003	RE
High quality studies only		0.5 (0.28–0.73)	95	13	< 0.0001	RE
FGF-23 levels difference between groups with and without arterial thickness	Pooled SMD	1.26 (0.36–2.17)	97	4	0.006	RE
CKD only		2.21 (0.5–3.91)	94	2	0.01	RE

*CKD* chronic kidney disease, *RE* random effect, *SMD* standardized mean difference.

**Table 3:** Summary of meta-analysis of Klotho with each sensitivity analysis.

Analysis and subgroup analysis	Effect measure	Pooled effect (95% CI)	I^2^ (%)	n	P value	Analysis model
Correlation between Klotho level and calcification score	Pooled r	− 0.388 (− 0.578 to − 0.159)	92	8	0.001	RE
CKD only		− 0.485 (− 0.658 to − 0.273)	90	6	< 0.0001	RE
Severe CKD only		− 0.523 (− 0.73 to 0.226)	91	4	0.001	RE
High quality studies only		− 0.159 (− 0.264 to − 0.05)	47	3	0.005	RE
Correlation between Klotho level and CIMT	Pooled r	− 0.38 (− 0.53 to − 0.207)	80	5	< 0.00001	RE
CKD only		− 0.26 (− 0.44 to − 0.07)	55	2	0.008	RE
High quality studies only		− 0.38 (− 0.55 to − 0.21)	42	3	< 0.0001	RE
Klotho levels difference between groups with and without arterial calcification	Pooled SMD	− 0.04 (− 0.33 to 0.24)	0	2	0.76	RE
Klotho levels difference between groups with and without arterial thickness	Pooled SMD	− 1.63 (− 3.11 to − 0.15)	97	3	0.03	RE
Without Castelblanco		− 2.27 (− 2.82 to − 1.72)	49	2	< 0.00001	RE

*CKD* chronic kidney disease, *RE* random effect, *SMD* standardized mean difference.

### Publication bias

Publication bias analysis using Funnel plot (Supplementary materials) indicates no publication bias for most analyses, except for pooled aOR of association between FGF-23 and arterial calcification in the linear regression model. However, after the study by Lee et al.^48^ was removed as an outlier, the funnel plot yielded a more symmetrical distribution without changing the pooled analysis. For analyses with a small number of included studies, publication bias analysis was not performed since the funnel plot and Egger’s test are not recommended for less than 10 studies^91^.

## Discussion

To the best of our knowledge, this study is the first meta-analysis that establishes the association of protein FGF-23 and Klotho with arterial calcification, thickness, and stiffness, and includes thorough sensitivity analyses. Our study indicates a significant positive correlation between FGF-23 and arterial calcification, CIMT, and PWV, and significant negative correlation between Klotho and arterial calcification and CIMT. FGF-23 and Klotho were also associated with arterial calcification. FGF-23 level was significantly higher in the groups with arterial calcification or thickness than in the group without arterial calcification or thickening. Furthermore, a significantly lower Klotho level was found in the arterial thickness group, not in the arterial calcification group, because only two studies were analyzed in the latter group.

As stated before, arterial thickness, calcification, and stiffness is a sequential process of arterial remodeling^1–5^. This sequential process is affected by the FGF-23/Klotho axis^14,15^. Although Klotho itself mainly acts as the cofactor of FGF-23, its expression is downregulated by FGF-23^19,92^. In the case of vascular Klotho deficiency, FGF-23 may induce the phenotype switching of contractile VSMCs to synthetic VSMCs mediated by FGF receptor-1 (FGFR-1) and Erk1/2 phosphorylation along with an increase in proliferation, which further induces thickening, and stiffening of the arterial wall^93^. This was confirmed in our study, which showed higher FGF-23, and lower Klotho levels in the arterial remodeling process. FGF-23 and Klotho also have contradictory effects on NO production. Klotho may revert the FGF-23-induced vasoconstriction by increasing NO production to dilate the arteries^93,94^. Furthermore, atherosclerotic plaques that reside in the arterial wall show a stronger FGFR signaling in response to FGF-23 and a lower expression of contractile VSMC phenotype^95^. The stronger FGFR signaling can cause further Klotho deficiency caused by FGF-23-induced Klotho downregulation. Interestingly, FGF-23, and Klotho have a unique or special affinity to FGFR-1^94,96^. The binding of Klotho to the principal effector site of FGFR-1 may induce the phosphaturic effects of FGF-23 on the kidney. Thus, the Klotho/FGFR-1/FGF-23 complex in the kidney is an important signaling pathway, either in generating, or counteracting hyperphosphatemia^94^. Hyperphosphatemia is avoided in this process because of its strong effect on inducing vascular calcification^97^. Therefore, all of these processes induce arterial remodeling, including vascular calcification, thickening, and stiffening.

Interestingly, the positive effect sizes of FGF-23 in vascular calcification and CIMT were stronger in the CKD-only subgroup analyses than in the overall analyses. Additionally, the pooled correlation between FGF-23 level and CIMT was also stronger in severe CKD only group than in all CKD group, albeit the number of studies was lower. This was further supported by a stronger negative correlation of Klotho to vascular calcification of the CKD-only study population; however, this was not seen in CIMT because only two studies analyzed Klotho in CKD. Despite these findings, we acknowledged that most of our included studies involved CKD patients. One could argue that there might be a tendency toward a significant finding, where higher FGF-23 and lower Klotho levels were associated with the conditions, due to the populations being predominantly CKD. Nevertheless, we observed that this is not utterly the case. For example, in the forest plot of the pooled correlation between FGF-23 and arterial calcification (Fig. 2A), studies with CKD and non-CKD-only populations presented with varying directions of effect sizes. Studies by Cianciolo^45^ and Nitta^30^ that included only CKD patients showed a negative direction of effect sizes. Meanwhile, studies by Masai^49^ and Morita^31^ showed a positive direction of effect sizes despite including non-CKD populations (suspected CAD patients). This finding was confirmed by our sensitivity analysis including only these two studies which still showed a significant positive effect size, although it was lower than that of the analysis with only CKD patients. In Klotho analyses, we could observe such similar cases, in which studies with non-CKD populations showed a negative direction of effect sizes, i.e., Koga^61^ and Morita^31^ in Fig. 2D and Jeong^63^, Keles^64^, and Keles^65^ in Fig. 2E. These findings indicated that FGF-23 and Klotho play important roles as a promoter and inhibitor, respectively^98^, in both CKD and non-CKD patients, and are not being entirely affected by kidney function status.

We also found a stronger FGF-23–CIMT correlation when two studies including children with CKD were excluded from the analysis. Two reasons could explain this interesting finding. First, despite having CKD, the pediatric populations were still in the growth and development phase, including their vascular thickness. The development of vascular thickness is ongoing throughout life; therefore, the vascular thickness might not be early seen^99^. Second, the number of children with CKD in the two studies was very limited compared with the number of adult patients in another five studies. Furthermore, the FGF-23–PWV correlation did not change much in the subgroup analyses excluding children and CKD-only participants. An interesting fact was stated by London^100^; i.e., the result of PWV measurement was age- and blood pressure-dependent. This might not change the correlation strength of FGF-23 and PWV because children and patients with CKD had an individual range of blood pressure.

Despite our findings, this study has four main limitations. First, the definitions, and parameters used for assessing arterial calcification, thickness, and stiffness vary. For example, several studies inappropriately analyzed arterial calcification using CIMT or PWV. CIMT was only designed for measuring the extent of the intimal and medial layers of the carotid arterial wall^101^, whereas PWV was only designed for measuring velocity and distensibility through the transmitted pulse wave in the arterial system^102^. Based on the latter statement, both CIMT and PWV did not measure the degree of calcification in the arterial wall, only the extent, and distensibility of the arterial wall, respectively. However, we overcame this limitation by classifying the analyses of calcification, thickness, and stiffness based on the assessment method used in each study: (1) calcification score to determine arterial calcification, (2) CIMT to determine arterial thickness, and (3) PWV to determine arterial stiffness. Second, the heterogeneities among the included studies were appreciable because of several factors, including study design, type of the analyzed artery, assessment process, sample size, age, and population type. We have also performed subgroup analyses to minimize the bias that might be caused by this limitation. We also have tried to explore the cause of the heterogeneity, i.e., measurement method used. However, all sample used blood specimen and almost all study used ELISA method. Hence, the heterogeneity might not likely be caused by the measurement method. Third, there was no detailed data regarding FGF-23 and Klotho levels in each CKD stage. There were limited studies which recruited participants from mild to moderate CKD only, since most included studies used HD or advanced stage CKD as their participants. Nevertheless, we have tried to do subgroup analysis for the available data to minimize this limitation, in which we proved that FGF-23 levels were significantly increased in arterial calcification, either in mild-to-moderate or severe CKD group. Lastly, considering that all included studies had an observational design investigating only associations, the true causality between FGF-23/Klotho and arterial calcification, thickness, and stiffness still cannot be discerned. Moreover, despite of the limitations, this meta-analysis could provide a useful insight on the role of FGF-23 and Klotho in arterial remodeling, since the underlying remodeling process is relatively complex and a unified conclusion is needed. Further research is warranted to establish the role of FGF23 and Klotho in clinical practice. We also suggest preclinical studies to explore further about the exact mechanism of FGF23 and Klotho on arterial remodeling process.

## Conclusion

The results of this meta-analysis confirmed the important roles of FGF-23 and Klotho in human arterial calcification, thickness, and stiffness, supporting their use as novel biomarkers for the early detection of arterial remodeling processes. Our study confirms that high FGF-23 levels and low Klotho levels are associated with arterial calcification, thickness, and stiffness, especially in patients with CKD. Despite the current findings, it is important to note that our included studies are mostly involved CKD patients. Hence, we encourage conducting further clinical studies to confirm diagnostic and prognostic roles of FGF-23 and Klotho in various populations, along with preclinical studies to establish the exact mechanism of both markers on arterial remodeling process.

### Supplementary Information

Supplementary Figure S1.

Supplementary Figure S2.

Supplementary Figure S3.

Supplementary Table S1.

Supplementary Table S2.

Supplementary Table S3.

Supplementary Table S4.

Supplementary Table S5.

## Data Availability

All data relating to the present study are available in this manuscript and supplementary files.
