# Supplementary figures and images for: Role of klotho and fibroblast growth factor 23 in arterial calcification, thickness, and stiffness: a meta-analysis of observational studies
Source: Sci Rep. 2024 Mar 8;14:5712. doi: 10.1038/s41598-024-56377-8 (PMC10923819; doi:10.1038/s41598-024-56377-8)

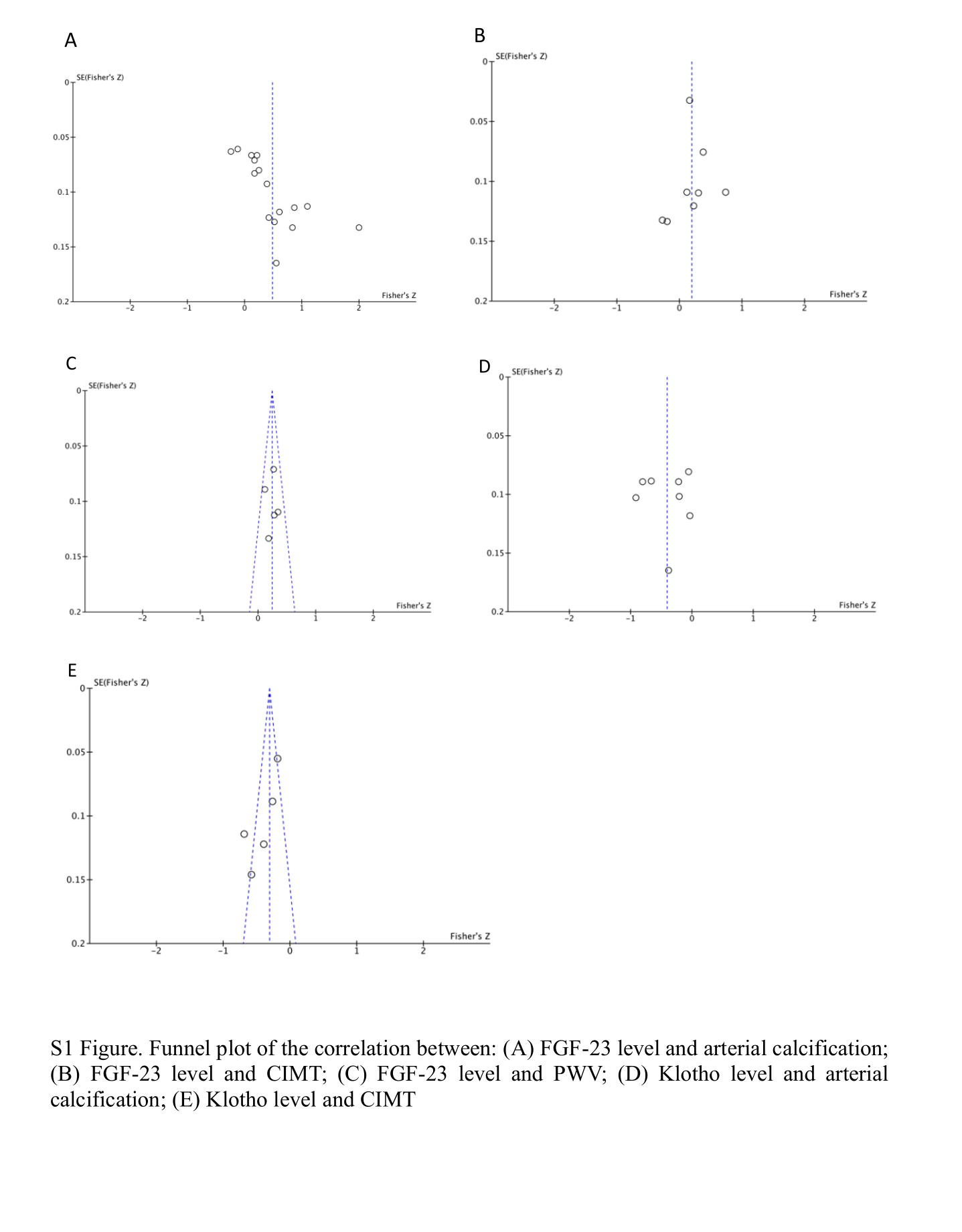

Supplement: Supplementary file 1 — Supplementary Figure S1. [file 41598_2024_56377_MOESM1_ESM.tiff]

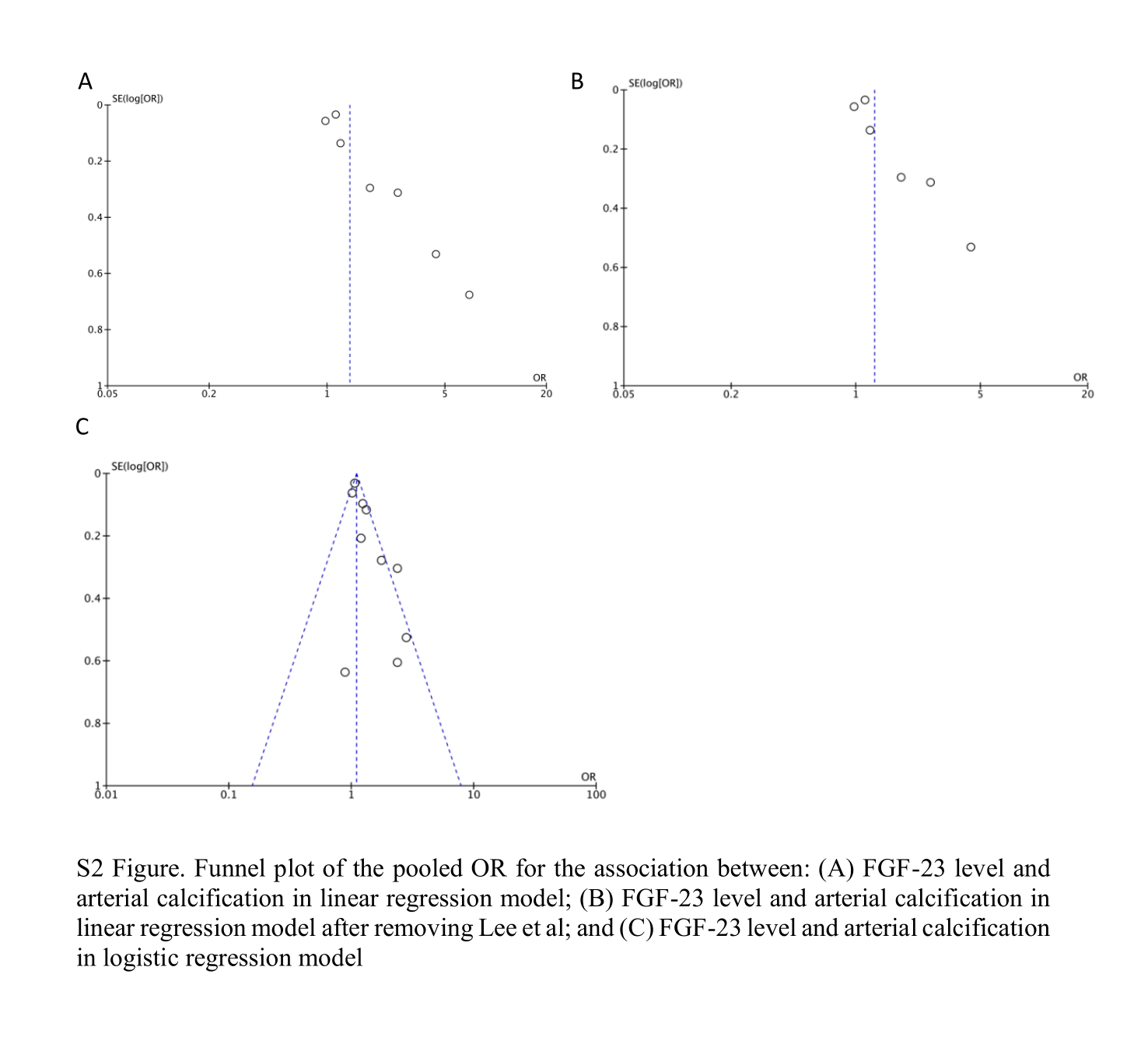

Supplement: Supplementary file 2 — Supplementary Figure S2. [file 41598_2024_56377_MOESM2_ESM.tiff]

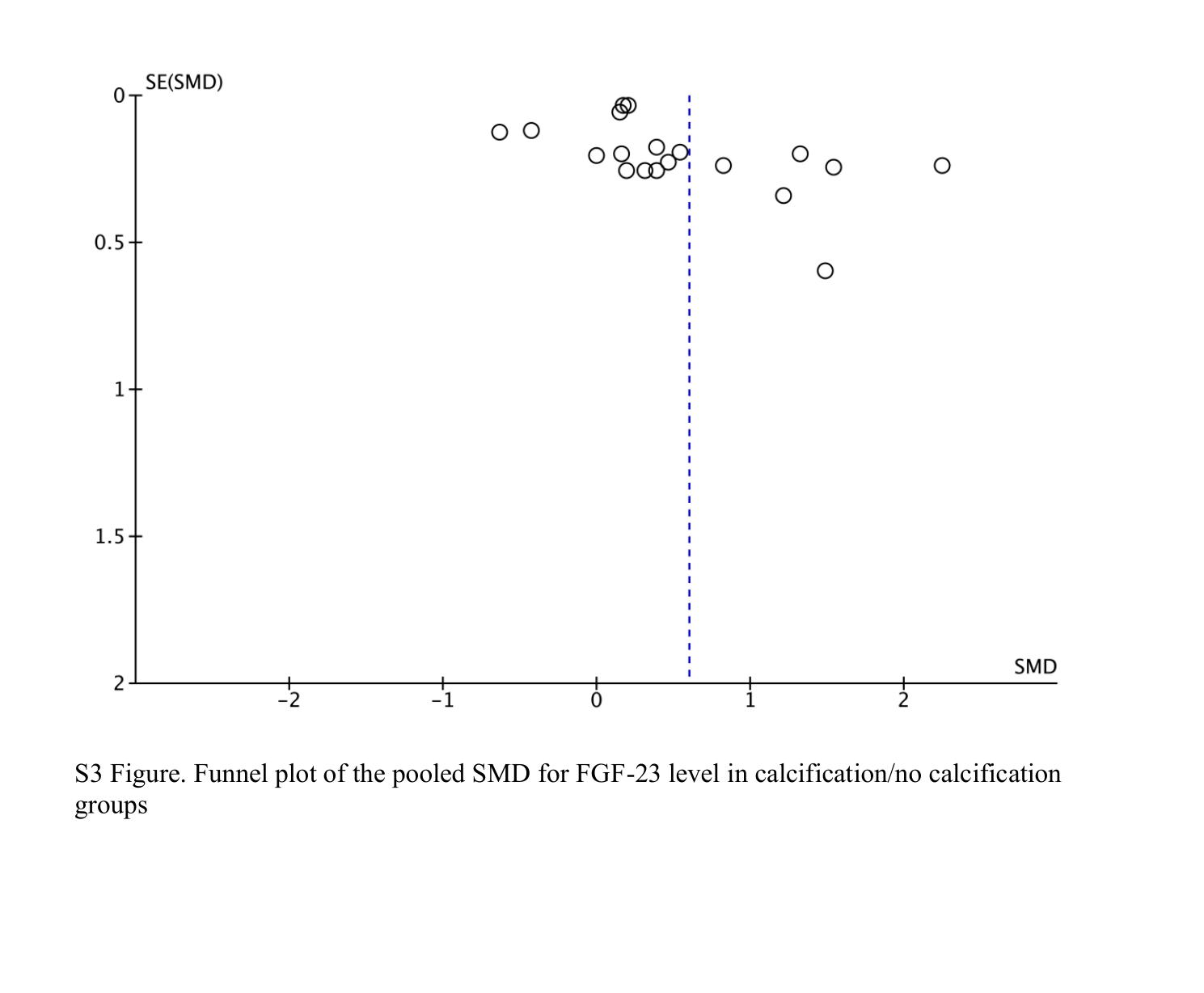

Supplement: Supplementary file 3 — Supplementary Figure S3. [file 41598_2024_56377_MOESM3_ESM.tiff]
