# Supplementary material for: Role of klotho and fibroblast growth factor 23 in arterial calcification, thickness, and stiffness: a meta-analysis of observational studies
Source: Sci Rep. 2024 Mar 8;14:5712. doi: 10.1038/s41598-024-56377-8 (PMC10923819; doi:10.1038/s41598-024-56377-8)
Supplement: Supplementary file 4 — Supplementary Table S1. [file 41598_2024_56377_MOESM4_ESM.docx]

**S1 Table.** Searching strategies to identify publications for our meta-analysis at different scientific repositories.

| Pubmed | #1 | (((((((((((klotho proteins[MeSH Terms]) OR (fibroblast growth factor 23[MeSH Terms])) OR (klotho[Title/Abstract])) OR (klotho protein*[Title/Abstract])) OR (KL protein*[Title/Abstract])) OR (KL[Title/Abstract])) OR (sKL[Title/Abstract])) OR (FGF23[Title/Abstract])) OR (FGF 23[Title/Abstract])) OR (fibroblast growth factor 23[Title/Abstract])) OR (fibroblast growth factor23[Title/Abstract])) OR (phosphatonin[Title/Abstract]) |
| --- | --- | --- |
|  | #2 | (((((((((((vascular calcification[MeSH Terms]) OR (coronary artery disease[MeSH Terms])) OR (vascular calcification*[Title/Abstract])) OR (coronary artery disease*[Title/Abstract])) OR (coronary arterial disease*[Title/Abstract])) OR (valvular calcification*[Title/Abstract])) OR (aortic calcification*[Title/Abstract])) OR (vascular stiffness[Title/Abstract])) OR (atherosclerotic plaque*[Title/Abstract])) OR (myocardial infarction*[Title/Abstract])) OR (vascular calcinos*[Title/Abstract])) OR (CAD[Title/Abstract]) |
|  | #3 | #1 AND #2 |
| Web of Science | #1 | (((((((((ALL=(Klotho protein*)) OR ALL=(klotho)) OR ALL=(fibroblast growth factor 23)) OR ALL=(fgf 23)) OR ALL=(KL protein*)) OR ALL=(KL)) OR ALL=(sKL)) OR ALL=(FGF23)) OR ALL=(Fibroblast growth factor23)) OR ALL=(phosphatonin) |
|  | #2 | (((((((((ALL=(vascular calcification*)) OR ALL=(coronary artery disease*)) OR ALL=(vascular stiffness)) OR ALL=(atherosclerotic plaque*)) OR ALL=(aortic calcification*)) OR ALL=(valvular calcification*)) OR ALL=(myocardial infarction*)) OR ALL=(vascular calcinos*)) OR ALL=(coronary arterial disease*)) OR ALL=(CAD) |
|  | #3 | #1 AND #2 |
| Scopus | #1 | (("klotho protein*") OR ("fibroblast growth factor 23") OR (klotho) OR ("KL protein*") OR (KL) OR (sKL) OR (FGF23) OR (FGF 23) OR ("fibroblast growth factor23") OR (phosphatonin)) |
|  | #2 | (("vascular calcification*") OR ("coronary artery disease*") OR ("coronary arterial disease*") OR ("valvular calcification*") OR ("aortic calcification*") OR ("vascular stiffness") OR ("atherosclerotic plaque*") OR ("myocardial infarction*") OR ("vascular calcinos*") OR (CAD])) |
|  | #3 | #1 AND #2 |
| EBSCO/CINAHL | #1 | (("klotho protein*") OR ("fibroblast growth factor 23") OR (klotho) OR ("KL protein*") OR (KL) OR (sKL) OR (FGF23) OR (FGF 23) OR ("fibroblast growth factor23") OR (phosphatonin)) |
|  | #2 | (("vascular calcification*") OR ("coronary artery disease*") OR ("coronary arterial disease*") OR ("valvular calcification*") OR ("aortic calcification*") OR ("vascular stiffness") OR ("atherosclerotic plaque*") OR ("myocardial infarction*") OR ("vascular calcinos*") OR (CAD)) |
|  | #3 | #1 AND #2 |
| Sciencedirect |  | ((klotho) OR (fibroblast growth factor 23) OR (KL protein) OR (FGF23) OR (FGF 23)) AND ((vascular calcification) OR (coronary artery disease) OR (valvular calcification) OR (aortic calcification)) |
