# Supplementary material for: Role of klotho and fibroblast growth factor 23 in arterial calcification, thickness, and stiffness: a meta-analysis of observational studies
Source: Sci Rep. 2024 Mar 8;14:5712. doi: 10.1038/s41598-024-56377-8 (PMC10923819; doi:10.1038/s41598-024-56377-8)
Supplement: Supplementary file 6 — Supplementary Table S3. [file 41598_2024_56377_MOESM6_ESM.docx]

**S3 Table.** Results of quality assessment of case-control studies based on the Newcastle-Ottawa Scale.

| **First author (year)** | **Selection** | | | | **Comparability** | **Exposure** | | | **Study quality** | |
| --- | --- | --- | --- | --- | --- | --- | --- | --- | --- | --- |
|  | **Adequateness of the case definition** | **Representativeness of the cases** | **Selection of controls** | **Definition of controls** | **Comparability of cases and controls on the basis of the design or analysis** | **Ascertainment of exposure** | **Same method of ascertainment for cases and controls** | **Non-response rate** | **Total score** | **Judgment** |
| Guo (2021)^31^ | * | * | 0 | 0 | 0 | * | * | 0 | 4 | Moderate |
