# Supplementary material for: Role of klotho and fibroblast growth factor 23 in arterial calcification, thickness, and stiffness: a meta-analysis of observational studies
Source: Sci Rep. 2024 Mar 8;14:5712. doi: 10.1038/s41598-024-56377-8 (PMC10923819; doi:10.1038/s41598-024-56377-8)
Supplement: Supplementary file 7 — Supplementary Table S4. [file 41598_2024_56377_MOESM7_ESM.docx]

**S4 Table.** Results of quality assessment of cohort studies based on the Newcastle-Ottawa Scale.

| **First author (year)** | **Selection** | | | | **Comparability** | **Exposure** | | | **Study quality** | |
| --- | --- | --- | --- | --- | --- | --- | --- | --- | --- | --- |
|  | **Representativeness of the exposed cohort** | **Selection of the non-exposed cohort** | **Ascertainment of exposure** | **Demonstration that outcome of interest was not present at start of study** | **Comparability of cohorts on the basis of the design or analysis** | **Assessment of outcome** | **Enough follow-up time length for outcome to occur** | **Adequacy of follow-up of cohorts** | **Total score** | **Judgment** |
| Bortnick (2019)^34^ | * | * | * | * | 0 | * | * | * | 7 | High |
| Buiten (2014)^75^ | * | * | * | * | ** | * | 0 | 0 | 7 | High |
| Di Lullo (2015)^55^ | * | * | * | * | * | * | 0 | 0 | 6 | Moderate |
| Ge (2022)^80^ | * | * | * | * | 0 | * | * | * | 7 | High |
| Krishnasamy (2017)^60^ | * | 0 | * | * | ** | * | * | * | 8 | High |
| Kurnatowska (2011)^63^ | * | * | * | * | 0 | * | * | * | 7 | High |
| Linefsky (2014)^64^ | * | * | * | * | 0 | * | * | * | 7 | High |
| Petrauskiene (2018)^32^ | * | * | * | * | 0 | * | * | * | 7 | High |
| Scialla (2013)^23^ | * | * | * | * | * | * | * | * | 8 | High |
| Zheng (2018)^22^ | * | * | * | * | * | * | * | * | 8 | High |
| Zhu (2019)^68^ | * | * | * | * | ** | * | * | * | 9 | High |
