# Supplementary material for: Role of klotho and fibroblast growth factor 23 in arterial calcification, thickness, and stiffness: a meta-analysis of observational studies
Source: Sci Rep. 2024 Mar 8;14:5712. doi: 10.1038/s41598-024-56377-8 (PMC10923819; doi:10.1038/s41598-024-56377-8)
Supplement: Supplementary file 8 — Supplementary Table S5. [file 41598_2024_56377_MOESM8_ESM.docx]

**S5 Table.** Results of quality assessment of cross-sectional studies based on the Newcastle-Ottawa Scale.

| **First author (year)** | **Selection** | | | **Comparability** | **Outcome** | | **Study quality** | |
| --- | --- | --- | --- | --- | --- | --- | --- | --- |
|  | **Representativeness of the sample** | **Sample size** | **Ascertainment of exposure** | **Comparability of subjects in different outcome groups on the basis of design or analysis** | **Assessment of outcome** | **Statistical test** | **Total score** | **Judgment** |
| Balci (2010)^49^ | 0 | 0 | ** | 0 | ** | * | 5 | Moderate |
| Baralic (2019)^43^ | * | 0 | ** | ** | ** | * | 8 | High |
| Bundy (2018)^50^ | * | * | ** | ** | ** | * | 9 | High |
| Cai (2015)^51^ | * | 0 | ** | ** | ** | * | 8 | High |
| Cancela (2012) | * | 0 | ** | ** | ** | * | 8 | High |
| Castelblanco (2022)^81^ | * | 0 | ** | 0 | ** | * | 6 | Moderate |
| Chen (2013)^20^ | * | 0 | ** | ** | ** | * | 8 | High |
| Cianciolo (2010)^40^ | * | 0 | ** | 0 | ** | * | 6 | Moderate |
| Coban (2018)^54^ | * | 0 | ** | 0 | ** | * | 6 | Moderate |
| Craver (2013)^41^ | * | 0 | ** | 0 | ** | * | 6 | Moderate |
| El Baz (2017)^21^ | * | 0 | 0 | 0 | ** | * | 4 | Moderate |
| Fayed (2019)^35^ | * | 0 | 0 | 0 | ** | 0 | 3 | Low |
| Figurek (2018)^61^ | * | 0 | ** | 0 | ** | * | 6 | Moderate |
| Ford (2011)^45^ | * | 0 | ** | 0 | ** | * | 6 | Moderate |
| Gutierrez (2009)^76^ | * | 0 | ** | 0 | ** | * | 6 | Moderate |
| He (2017)^44^ | * | 0 | ** | 0 | ** | * | 6 | Moderate |
| Ibrahim (2018)^79^ | * | 0 | ** | 0 | ** | * | 6 | Moderate |
| Jasani (2018)^56^ | * | 0 | ** | 0 | ** | * | 6 | Moderate |
| Jeong (2013)^57^ | * | 0 | ** | ** | ** | * | 8 | High |
| Keles (2015)^59^ | 0 | 0 | ** | 0 | ** | * | 5 | Moderate |
| Keles (2016)^53^ | 0 | 0 | ** | 0 | ** | 0 | 4 | Moderate |
| Koga (2021)^48^ | 0 | 0 | ** | 0 | ** | * | 5 | Moderate |
| Lee (2016)^78^ | 0 | 0 | ** | ** | ** | * | 7 | High |
| Lin (2022)^77^ | * | 0 | ** | 0 | ** | * | 6 | Moderate |
| Masai (2013)^52^ | * | 0 | ** | ** | ** | * | 8 | High |
| Milovanova (2022)^38^ | * | 0 | ** | 0 | ** | * | 6 | Moderate |
| Morita (2015)^65^ | * | 0 | ** | ** | ** | * | 8 | High |
| Mudi (2019)^46^ | * | 0 | ** | 0 | ** | * | 6 | Moderate |
| Muzasti (2021)^42^ | * | 0 | 0 | 0 | ** | * | 4 | Moderate |
| Nakayama (2013)^66^ | * | 0 | ** | ** | ** | * | 8 | High |
| Nasrallah (2010)^71^ | * | 0 | ** | ** | ** | * | 8 | High |
| Nitta (2018)^62^ | * | 0 | ** | ** | ** | * | 8 | High |
| Ortiz (2020)^47^ | * | * | ** | 0 | ** | * | 7 | High |
| Pencak (2013)^36^ | * | 0 | ** | 0 | ** | * | 6 | Moderate |
| Salam (2020)^29^ | * | 0 | ** | 0 | ** | * | 6 | Moderate |
| Sandoval (2015)^33^ | * | * | ** | 0 | ** | * | 7 | High |
| Schoppet (2012)^67^ | * | * | ** | ** | ** | * | 9 | High |
| Srivaths (2014)^37^ | 0 | 0 | ** | 0 | ** | * | 5 | Moderate |
| Singh (2022)^25^ | * | 0 | ** | 0 | ** | * | 6 | Moderate |
| Tarigan (2019)^74^ | * | * | ** | 0 | ** | * | 7 | High |
| Turan (2016) | * | 0 | ** | ** | ** | * | 8 | High |
| Villodres (2019)^58^ | * | 0 | ** | 0 | ** | * | 6 | Moderate |
| Yilmaz (2015)^30^ | * | 0 | ** | 0 | ** | * | 6 | Moderate |
| Yu (2018)^39^ | * | 0 | ** | 0 | ** | * | 6 | Moderate |
| Zaki (2018)^73^ | * | 0 | 0 | 0 | ** | * | 4 | Moderate |
| Zamparini (2018) | 0 | 0 | ** | 0 | ** | * | 5 | Moderate |
| Zayed (2015)^72^ | * | 0 | ** | 0 | ** | * | 6 | Moderate |
| Zeng (2015)^69^ | * | 0 | ** | 0 | ** | * | 6 | Moderate |
| Zhang (2015)^70^ | * | 0 | ** | 0 | ** | * | 6 | Moderate |
| Zhu (2023) | * | 0 | ** | ** | ** | * | 8 | High |
